# The Conundrum of Spinal Metastases—A Review of Current Management

**DOI:** 10.3390/jcm14207279

**Published:** 2025-10-15

**Authors:** Bogdan Florin Iliescu, Daniel Ilie Rotariu, Loredana Mariana Agavriloaei, Bogdan Costachescu

**Affiliations:** 1 Department of Neurosurgery, Grigore T Popa University of Medicine and Pharmacy Iasi, 11 Universitatii St, 700115 Iasi, Romania; bogdan-florin.iliescu@umfiasi.ro (B.F.I.); mariana-loredana_curecheriu@umfiasi.ro (L.M.A.); bogdan.costachescu@umfiasi.ro (B.C.); 2 “Prof Dr N Oblu” Clinical Emergency Hospital Iasi, 2nd Ateneului St, 700309 Iasi, Romania

**Keywords:** spinal metastasis, neuro-oncology, minimally invasive spinal surgery (MISS)

## Abstract

Spinal metastasis remains a significant clinical issue, frequently resulting in substantial pain and disability among cancer patients. Conventional management strategies have historically included chemotherapy, radiotherapy, and open surgical intervention. However, advancements in minimally invasive spinal surgery (MISS) have notably shifted the therapeutic landscape. This review examines recent evidence surrounding MISS, directly comparing it to traditional open procedures. Current literature demonstrates that MISS typically results in reduced intraoperative blood loss, shorter hospitalization durations, decreased infection rates, and functional outcomes that are at least equivalent—if not superior—to those of open surgery. Additionally, the emergence of hybrid therapeutic approaches—specifically, the utilization of separation surgery followed by stereotactic radiosurgery—has shown promise in achieving local tumor control, particularly in select malignancies. This narrative review also evaluates contemporary clinical decision-making frameworks such as NOMS, LMNOP, and NESMS. Further, it advocates for the integration of advanced prognostic tools and tumor genomics to enable more personalized treatment strategies for individuals with spinal metastasis.

## 1. Introduction

Spinal metastases present a significant clinical challenge for cancer patients. Approximately 70% of individuals diagnosed with cancer will develop metastatic lesions in the spine, and among these, 10–20% experience complications such as pain, neurological deficits, and a substantial decline in quality of life [1]. With improvements in cancer survival rates and the overall rise in cancer prevalence, the incidence of spinal metastases has likewise increased [2]. Certain malignancies are particularly prone to metastasizing to bone. Breast and lung cancers are the most frequently implicated, with prostate, lymphoma, kidney, gastrointestinal tract cancers, and melanoma following. The thoracic spine is the most common site of involvement, but the lumbar region is also affected.

The primary aims of surgical intervention for spinal metastases are pain relief and the preservation or restoration of ambulatory function, both of which are critical for maintaining quality of life. Management is multidisciplinary, typically involving a combination of chemotherapy, radiation therapy, and surgery [3]. To address the diverse responses of cancer patients to treatment, a comprehensive approach that combines chemotherapy, radiation, and surgery is utilized for the treatment of metastatic spine cancers [4]. Various clinical teams have developed decision-making algorithms to guide treatment, though recent advancements in surgical techniques and a deeper understanding of tumor biology have prompted further refinement of these frameworks [5]. Minimally invasive spine surgery (MISS) has become increasingly prominent, initially for degenerative spinal disease and now as a viable or even preferable option for metastatic spinal involvement. Emerging evidence supports the use of hybrid approaches that combine MISS techniques with stereotactic radiosurgery (SRS), showing promising outcomes compared to traditional open procedures.

## 2. Minimally Invasive Spine Surgery (MISS)

MISS (minimally invasive spinal surgery) and minimal-access spinal technologies (MAST) are surgical procedures developed to reduce operative-related injuries and postoperative complications.

The main procedures used in metastatic spinal surgery include small incision decompression, percutaneous pedicle screw fixation, minimally invasive separation surgery, percutaneous vertebroplasty (PVP) or kyphoplasty (PKP), endoscopic-assisted spine surgery, radiofrequency ablation, laser interstitial thermal therapy (LITT), and radioactive seed implantation [6].

Minimally invasive—or minimal-access—surgical approaches for spinal metastases can significantly accelerate postoperative recovery and reduce surgical complications. This is particularly advantageous for oncology patients, as it enables them to promptly resume their cancer therapies. For anterior procedures, prior research has provided tubular retractor systems and thoracoscopic aid [7,8], minimally invasive techniques for decompression [9,10], and corpectomy for posterior surgeries. In spinal metastasis surgery, other MIS techniques such as image-guided stereotactic navigation systems and percutaneous pedicle screw fixation are also useful therapeutic modalities [11,12].

Previous studies have indicated that minimally invasive surgery (MIS) is associated with fewer surgical site complications and similar surgical outcomes compared to traditional open surgery [13,14]. More recently, a systematic review encompassing 26 studies suggested that MIS may reduce the incidence of surgical site infections, decrease hospital stays, and limit blood loss in patients undergoing treatment for spinal metastases—all without sacrificing the precision of instrument placement or compromising overall patient outcomes [15]. Table 1 summarizes the advantages and disadvantages of the reviewed data.

### 2.1. Small Incision Decompression

Small incision decompression is generally preferred for procedures involving one or two spinal levels, mainly due to the limited surgical exposure it provides. Typically, a short midline incision is utilized for posterior resections, whereas a paramedian approach is chosen for facet joint resections. When feasible, surgeons aim to remove as much of the ventrally located tumor as possible, carefully creating a margin around the dura mater. This approach is intended to facilitate subsequent adjuvant radiotherapy, like the principles applied in separation surgery.

Separation surgery essentially entails creating a physical buffer between the spinal cord and the tumor to facilitate safe delivery of stereotactic radiosurgery (SRS) [16]. The procedure involves a circumferential, 360-degree decompression of the spinal cord, allowing the dural sac to expand fully. Surgeons aim to establish at least a 2–3 mm gap between the tumor and the cord, ensuring that the spinal cord is adequately protected and that SRS can be administered without undue risk. While the approach is assertive, it is a necessary measure to optimize outcomes in spinal tumor management. The posterolateral transpedicular approach, as reported by Bilsky et al. [17], is a safe, efficient, and adaptable surgical technique for circumferential spinal cord decompression, among other techniques.

Ventral decompression stands out as both the most technically demanding and pivotal step in separation surgery. Intraoperative confirmation of adequate decompression is essential, and surgeons rely on it to ensure the procedure’s effectiveness. Achieving sufficient ventral decompression has been clearly associated with improved long-term local control, underscoring its significance in surgical outcomes [18]. 20% of the posterior vertebral body must typically be removed following posterior longitudinal ligament excision to achieve sufficient ventral decompression [16]. A stereotactic navigation system or ultrasonography can be used to validate ventral decompression intraoperatively [19].

When paired with SRS, separation surgery is the preferred treatment for patients with high-grade spinal cord compression of radioresistant tumors, according to interdisciplinary decision-making systems such as the NOMS framework [20]. Several studies [12,21] have reported favorable outcomes with the hybrid approach—performing separation surgery first, followed by SRS. This combination appears to be effective based on current evidence.

### 2.2. Percutaneous Pedicle Screw Fixation

The surgical procedure can be used alone or in combination with small incision decompression, as mentioned. Percutaneous pedicle screw fixation is generally recommended in patients with instability. Cemented pedicle screws are also an option for further reinforcement [13]. This technique does present notable limitations. The cannulated screws used here are larger in diameter, which restricts their application to only the lower thoracic and lumbar vertebrae [6].

### 2.3. Percutaneous Vertebroplasty (PVP) or Kyphoplasty (PKP)

Injection of PMMA (percutaneous polymethylmethacrylate) or by balloon insertion and inflation (kyphoplasty) are techniques that reinforce the bone, supporting the anterior column. There are studies that show that PMMA has antitumor activity and provides pain relief by stabilizing the microfractures [22]. Injections of cement alone are mainly used in cases with anterior vertebral body involvement. When the lesion affects the posterior column, a combination of pedicle screws is required to increase the stability of the construct [6]. Usually, the technique is used to first do a biopsy for tissue diagnosis, then inject for pain relief and reinforcement [23].

### 2.4. Endoscopy-Assisted Spine Surgery

Endoscopy-assisted spine surgery has limited indications because of its narrow operative field. Recent case studies have documented the application of the endoscopic technique and percutaneous endoscopic interlaminar decompression in the treatment of spinal metastatic tumors. The authors mentioned the shortcomings of using an endoscope, which were a narrow operating space, difficult hemostasis, and a long learning curve [23,24,25]. Other case reports mention using the endoscopy-assisted posterolateral approach and anterior reconstruction of the anterior column with open surgery [26].

### 2.5. Radiofrequency Ablation

Radiofrequency ablation (RFA) is a palliative procedure for spinal metastases. It is a minimally invasive, image-guided procedure through which a transpedicular bipolar electrode that generates high-frequency alternating current produces controlled heat with necrosis and cell death [27].

Globally, this procedure reduces local pain, improves the neurological status, reduces the risk of fracture on pathological bone (through vertebroplasty performed at the end of the ablative procedure), and increases the quality of life of the patients [28].

Lu and colleagues conducted a comprehensive review outlining when it makes sense to use RFA in combination with vertebroplasty, and when it is an absolute no-go. The procedure is generally recommended for patients struggling with pain from spinal metastases, especially when there is an associated soft tissue mass or a looming risk of pathological fracture. It is also indicated for individuals whose pain is just not responding to standard painkillers and is seriously impacting their quality of life. Even when the pain is moderate but persistent, as long as the clinical symptoms match up with imaging findings, this approach can be considered for palliative care [28].

On the other hand, there are clear situations where this treatment should be avoided. These include any presence of local or systemic infection, severe coagulopathy, tumor-related compression of the spinal cord, or outright spinal instability. In such cases, the risks clearly outweigh any potential benefits. [28].

### 2.6. Laser Interstitial Thermotherapy (LITT)

Laser Interstitial Thermotherapy (LITT) is a valuable tool in the management of spinal metastases, offering a minimally invasive option for tumor ablation with benefits in pain relief, preservation of spinal stability, and compatibility with other therapies. LITT stands out for its remarkable precision and shorter recovery periods, making it a valuable option, especially for patients who are not suitable candidates for more invasive procedures. It is an important addition to the range of treatments for spinal metastatic disease. When tumor growth leads to concerns about spinal stability, LITT can reduce tumor volume, which may help preserve the structural integrity of the spine and lower the risk of fractures. This method is not limited to solo use; it is often combined with other therapies like radiation or chemotherapy to potentially improve outcomes. Plus, it can be used alongside vertebral augmentation techniques, such as vertebroplasty or kyphoplasty, to further stabilize the spine after ablation [29]. In a recent review, Cardia et al. found that LITT is safe and provides effective local control for epidural compression from metastases, particularly in the thoracic spine. The authors propose considering LITT as an alternative to open surgery in selected patients with spinal metastases [30].

### 2.7. Radioactive Seed Implantation

Radioactive seed implantation represents a minimally invasive approach in which a small, synthetic radioactive iodine seed is directly inserted into the tumor. This method continuously delivers localized radiation, maximizing the dose to the tumor itself while minimizing the risk of harm to surrounding structures such as the spinal cord or nerve tissue. The technique may be applied independently or in conjunction with procedures like percutaneous vertebroplasty (PVP) or percutaneous kyphoplasty (PKP). Notably, research by Yang et al. demonstrated that combining radioactive seed implantation with PVP significantly alleviates pain, enhances spinal stability, improves patients’ quality of life, and lowers the risk of paraplegia in individuals suffering from spinal metastases [31].

## 3. Comparison Between Open and MISS Techniques

In Scaramuzzo et al., a level IV evidence study, percutaneous osteosynthesis (without decompression) was compared with open posterior fusion; patients requiring extensive decompression were excluded from the MISS cohort. However, more recent series demonstrate that MISS techniques are feasible for one- to two-level decompression and for minimally invasive separation surgery, particularly when the goal is limited neural decompression and early return to adjuvant therapy. When decompression involves more extensive ventral tumor removal or multilevel exposure, an open approach remains indicated.

Overall, MISS consistently results in reduced intraoperative blood loss, fewer transfusion requirements, diminished surgical-site infection risk, and shorter hospital stays, while maintaining comparable neurological and functional outcomes. These advantages make MISS particularly beneficial for frail oncology patients and those who must resume systemic therapy promptly. The main limitation of the study is that the indications apply to patients who do not need spinal decompression [32].

Even though the sample size was relatively low for the presented study, the impact on mortality and morbidity was statistically significant. The results are in accordance with other similar studies [6] that are more in favor of minimally invasive techniques for patients with spinal metastasis.

Pranata et al. conducted a meta-analysis comparing minimally invasive spine fusion to the traditional approach, analyzing eight studies with a total of 486 patients. Their level II evidence meta-analysis paper echoed those of Scaramuzzo et al., reporting reductions in blood loss, transfusion rates, and length of hospital stay for the minimally invasive group. Notably, the minimally invasive technique also demonstrated a lower rate of surgical site infections [33]. Supporting this, Lu et al. reported a 1% infection rate for minimally invasive surgery, compared to 4% for open procedures [34]. All the recently published data summarized in Table 2 suggest that MISS is potentially superior compared to classic techniques.

## 4. Hybrid Therapy

Hybrid therapy refers to a treatment approach that combines separation surgery with stereotactic radiosurgery. In separation surgery, the surgeon does not attempt a complete tumor removal—instead, the main goal is to decompress the spinal cord by establishing a clear space between the cord and the tumor. This decompression creates a safer and more effective target zone for subsequent stereotactic radiosurgery, which delivers highly focused radiation to the tumor while minimizing risk to the spinal cord. The benefits of this combined strategy were first highlighted in 2005, following the influential prospective randomized trial conducted by Patchell and colleagues [35]. The new advancements in minimally invasive surgery described enable more patients to tolerate decompression followed by radiation treatment.

Several recent single-center retrospective studies have demonstrated the effectiveness of this procedure, with patient follow-up extending to two years. For instance, Chakravarthy et al. reported that individuals with metastatic epidural spinal cord lesions—stemming from either colorectal cancer or non-small cell lung cancer—who underwent a hybrid therapy protocol achieved promising results. Specifically, at the two-year follow-up after surgery, local control rates were 86.7% for colorectal cancer patients and 95% for those with non-small cell lung cancer [36,37].

Kang et al. analyzed 13 studies in a recent meta-analysis with a total of 661 patients involved who had metastatic epidural spinal cord lesions and were treated with hybrid therapy. The results showed that the local progression rate was 10.2% at 1 year and 13.7% at 2-year follow-up [37]. The positive outcomes of hybrid therapy prevent spine surgeons from operating on patients with spinal metastases that carry a high risk of surgical morbidity during en bloc resections [38].

A recent review analyzes the trends that emerge in radiation therapy, involving the superiority of stereotactic body radiation therapy (SBRT) versus conventional external beam radiation therapy (cERBT), which offers a more targeted therapy with superior rates of preserved ambulation [39].

The 2024 ESTRO clinical practice guideline formally supports widely used high-dose SBRT regimens for intact vertebral lesions—examples include 24 Gy in a single fraction, 20 Gy × 1, 2 × 12 Gy, 3 × 10 Gy, and 5 × 7 Gy—while emphasizing strict adherence to spinal cord and thecal sac dose constraints. After separation surgery, which physically displaces tumor from the spinal cord, the guidelines underscore the necessity of confirming sufficient target-to-cord distance prior to SBRT delivery to minimize neurological risk [40]. When considering re-irradiation, the guidelines recommend cumulative dose-summation and interval-adjusted spinal cord EQD2 limits. Retreatment remains feasible, provided that single-fraction doses are reduced or hypofractionated schedules are selected, and that prior cord exposure is respected [40]. Collectively, these updates reinforce the clinical rationale for minimally invasive separation techniques, as they facilitate safe delivery of ablative SBRT while prioritizing spinal cord protection.

MISS stabilization without decompression is most appropriate when the mechanical instability drives symptoms rather than cord compression or significant epidural disease. Ideal candidates for this technique would have a SINS ≥ 13 (frank instability) or painful pathologic fractures causing mechanical pain; patients with a Bilsky Grade 0–1a, i.e., epidural disease without thecal sac compression; patients that are neurologically intact or show minimal deficits (e.g., radicular pain only); patients with radio responsive tumors (e.g., breast, prostate, myeloma, lymphoma), where SBRT or conventional RT alone can achieve tumor control; patients with limited prognosis or poor systemic reserve, where decompression risk outweighs benefit. MISS fixation stabilizes the spine with minimal morbidity, reduces pain, and allows early mobilization and rapid resumption of systemic therapy. In these cases, radiation alone addresses tumor burden, while MISS stabilization prevents mechanical progression (e.g., lytic thoracic metastasis from breast cancer (Bilsky 1a, SINS 13) → MISS percutaneous cement-augmented fixation + SBRT within one week).

Modern consensus (21, 39) supports early, coordinated delivery of SBRT after surgery once wound healing is adequate:

- Within 2–4 weeks post-separation surgery. Recent evidence suggests that this period creates an optimal balance between wound healing and minimizing risk of local progression. Local control ≥ 85–95% at 1 year (21).
- <2 weeks—feasible for small, well-healed incisions (MISS separation); early SBRT minimizes risk of tumor regrowth. Used increasingly with minimally invasive approaches.
- >6 weeks delay—associated with worse local control (tumor repopulation and epidural recurrence). Avoid unless medically necessary.

The key principle to be followed is that SBRT should follow separation surgery as soon as wound integrity allows, ideally 7–21 days postoperatively in MISS cases.

Table 3 summarizes the plan changes that are dictated by the Bilsky grade and radio responsiveness.

## 5. Surgical Indications Based on Decision-Making Algorithms

Surgical indications for spinal metastases are classified as definitive or relative. The primary goals are pain control, preservation of neurological function, and mechanical stabilization. Urgent decompression is required in patients presenting with clinical myelopathy due to metastatic disease, with evidence showing improved outcomes when performed within 24 h of symptom onset.

The Spinal Instability Neoplastic Score (SINS) is widely used to assess mechanical stability and guide decisions about spinal fixation. A SINS of 13 or higher strongly supports surgical stabilization, whereas scores of 0–6 indicate stability and are typically managed non-operatively. For intermediate scores (7–12), where instability is indeterminate, management should be individualized and decisions are best made through a multidisciplinary discussion that considers tumor biology, prognosis, neurological status, and overall patient fitness.

Relative indications include selected cases of oligometastatic, radioresistant tumors and symptomatic radiculopathy due to nerve root compression. Table 4 summarizes the definitive and relative indications for surgical treatment.

Patients with spinal metastasis truly benefit from a team-based approach, involving various specialists—oncologists, surgeons, and often pain management experts—all collaborating to create an individualized treatment plan. In practice, decision-making is not arbitrary; clinicians usually rely on established frameworks. Some of these are classification-based, focusing on specific categories and criteria, while others are principle-based, emphasizing core guidelines for care. Ultimately, there is no universal solution—these algorithms provide structured, yet adaptable strategies tailored to each patient’s unique situation.

### 5.1. Classification-Based Algorithms

The indication for surgery is primarily dependent on a minimum of 3 months of life expectancy; therefore, classifications such as Tomita [41], Tokuhashi [42], Bauer [43], and Katagiri [44] scoring systems were used to evaluate longevity of a newly diagnosed patient.

Recent developments in oncological therapy with molecular targeted therapies, immunotherapy, and hormonal therapies influence the survivorship of these patients, and the need for a new updated system appeared [45]. The New England Spinal Metastasis Score (NESMS) was introduced in 2015 [46] and comprises a modified Bauer score, serum albumin level, and ambulatory state (Table 5). The scoring system was created by the authors using data from multiple institutions, and it has been verified both retrospectively [47] and prospectively [48] as a valid and trustworthy tool for spinal metastatic prediction. The authors of a recently published prospective study found that the NESMS significantly outperformed the standard scoring methods (Tokuhashi, Tomita, and Spinal Instability Neoplastic Score [SINS]) in differentiating patient survival [49].

A considerable number of researchers have begun implementing advanced technologies—particularly AI algorithms—when developing decision-making systems. For instance, the Skeletal Oncology Research Group (SORG) conducted a study involving 649 patients, comparing projected survival outcomes using traditional methods, nomograms, and modern boosting algorithms. This approach reflects the increasing integration of innovative computational techniques in medical research [50]. The authors of the study noted that their nomogram was both accurate and user-friendly. More recently, a “second-generation model” was introduced by SORG, incorporating machine-learning techniques to develop a novel prognostic tool for spinal metastases.

### 5.2. Principle-Based Algorithms

Based on the literature review [45] and our experience, it is not overstated to say that principle-based classifications offer more detailed therapy recommendations for each patient with spinal metastases based on their oncologic, systemic, and functional condition. Furthermore, compared to classification-based prognostic models, these systems may more accurately represent developments in systemic, radiation, and surgical treatments, such as molecular target therapy, SRS, and separation surgery.

The NOMS framework—which stands for neurologic, oncologic, mechanical, and systemic—was first introduced in 2006 as a principle-based decision-making model. For the neurologic (N) element, clinicians assess spinal cord involvement using the Bilsky grading scale [51]. This approach used the Bilsky grade [17] to evaluate the neurologic (N) component. While the mechanical (M) component is calculated by evaluating the spinal column stability using the SINS, the oncologic (O) component is based on the anticipated response to existing treatments, notably radiotherapy. Regardless of other factors, this could help surgeons decide whether to do surgical stabilization [52]. Ultimately, the patient’s ability to tolerate the recommended course of treatment is determined by the systemic (S) component. Based on these four factors, the best course of action for each patient with spinal metastases is recommended, including cutting-edge techniques like SRS and separation surgery [1].

Paton and colleagues introduced the LMNOP system—yes, the acronym is intentionally reminiscent of the alphabet—to serve as an alternative to the traditional NOMS framework. This system incorporates several domains: spine (L), mechanical instability (M), neurology (N), oncology (O), and patient fitness and prognosis, as well as the patient’s response to prior therapy (P). Notably, LMNOP expands on previous models by including both the quantity and anatomical location of metastatic spinal lesions, alongside an explicit evaluation of how patients responded to earlier systemic treatments or radiation. This approach allows clinicians to individualize treatment strategies by considering prior therapeutic outcomes, potentially offering a more nuanced and precise method for decision-making in the management of spinal metastases [53].

Decision-making algorithms in the management of spinal metastases are crucial for optimizing patient care. These algorithms provide structured and evidence-based approaches to evaluating and treating patients with spinal metastases, which is a complex and often urgent clinical scenario. Algorithms help ensure that all patients receive standardized care based on the best available evidence, reducing variability in treatment approaches among different clinicians and institutions. They provide a systematic approach to quickly assess the patient’s condition, which is crucial in urgent cases where spinal cord compression or instability might be present. They help balance the potential risks and benefits of different treatment modalities based on patient-specific factors. They assist in identifying high-risk patients who might need more aggressive interventions or closer monitoring, thereby preventing complications such as spinal cord compression or pathological fractures. Therefore, these algorithms enhance clinical decision-making, promote multidisciplinary coordination, optimize treatment selection, and improve patient outcomes.

**Figure 1:**
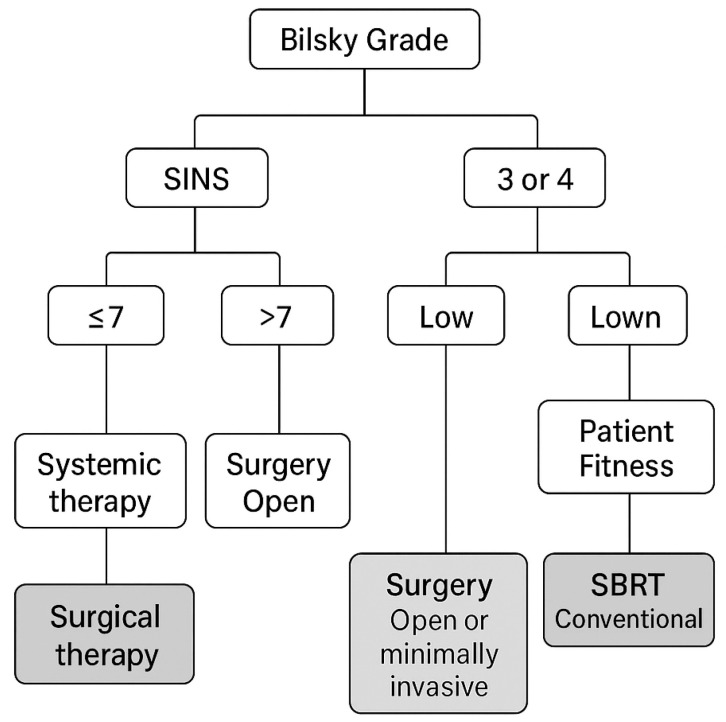
Algorithm for the management of spinal metastases.

In conclusion, the current MISS literature is limited by small, retrospective series with heterogeneous indications (fixation vs. decompression), varied surgical techniques, and non-standardized oncologic co-interventions. Outcome definitions and follow-up duration are inconsistent, and patient selection strongly favors MISS in fitter or less complex cases. Collectively, these limitations reduce the robustness of conclusions, particularly regarding survival or neurologic outcomes. Given the amount of data that we have available we believe that an algorithm that combines Bilsky grade, SINS, tumor histology radiosensitivity, and patient fitness to guide the recommended action can be devised and we present it in Figure 1.

Furthermore, we devised a technique matrix that specifies when to use MISS decompression, cement-augmented screws, RFA or LITT, and brachytherapy seeds, and list key contraindications for each option, as seen in Table 6.

## 6. Prospective Trends and Future Directions

With the rapid developments in cancer genetics and the introduction of innovative treatment options, it is clear that current prognostic models for metastatic spinal cancers are due for a serious update. The most recent literature points to a few key recommendations for future decision-making systems. First, these systems should be developed and validated using an international or multi-institutional database. Second, tumor genetics needs to be front and center in the decision process; it is far too important to overlook. Third, it makes sense to incorporate advanced methods, like artificial intelligence, to improve accuracy and adaptability. And finally, these prognostic models should be integrated with principle-based decision-making frameworks to ensure that the guidance they offer is both data-driven and clinically relevant [23,54].

Future systems for making decisions ought to incorporate both principle- and classification-based approaches. While principle-based systems choose the best course of action based on these survival estimates, classification-based systems, also known as prognostic models, predict the patient’s remaining survival. Modern decision-making systems ought to integrate these two systems and simultaneously evaluate the remaining survival and the best course of action [39].

The management of spinal metastases is a complex, multidisciplinary process aimed at improving patient outcomes, including pain relief, preservation or restoration of neurological function, and maintaining quality of life [6]. Based on the current consensus and available therapies, a couple of key points are essential for proper management starting with a multidisciplinary approach. Effective management involves a team of specialists including oncologists, radiologists, neurosurgeons, orthopedic surgeons, pain specialists, and rehabilitation professionals. Collaborative decision-making is crucial for optimizing treatment outcomes.

Minimally invasive surgical (MIS) approaches have significantly improved patient outcomes, offering reduced operative morbidity, faster recovery, and less postoperative pain. Surgery is particularly considered for patients with spinal instability, significant neurological deficits, or intractable pain that is not responsive to other treatments. Advances in surgical techniques, including MIS, have greatly improved outcomes and reduced recovery times [34].

Radiation therapy is the cornerstone in the management of spinal metastases, especially for pain relief and local control of the disease. Techniques like stereotactic body radiotherapy (SBRT) specifically target lesions with minimal damage to surrounding tissues [39]. Advances in diagnostic methods and treatment options (both surgical and non-surgical), growing experience, and an increasing body of clinical data make this field a continuously evolving one requiring frequent updates and appropriate changes in the approach of patients with spinal metastases. Hybrid therapy, which combines MIS with stereotactic radiotherapy, has recently become increasingly popular.

The future of managing spinal metastases through MISS involves several advancements and areas of research. Integration of advanced imaging techniques, such as high-resolution imaging and real-time intraoperative navigation systems, can enhance the precision of MISS, making procedures safer and more effective [55]. Robotic-assisted surgery is another promising area, providing greater accuracy and control during minimally invasive procedures, reducing surgeon fatigue, and improving patient outcomes [56]. Still, there is a significant amount of data that could be obtained from standardized prospective trials Comparing MISS vs. open surgery. Most available data we have available are retrospective and heterogeneous in patient selection, disease burden, and adjuvant therapy use. Prospective, multicenter studies are needed to define patient selection criteria for MISS decompression and stabilization, quantify quality-of-life, cost-effectiveness, and long-term local control outcomes, and evaluate outcomes by tumor histology and extent of epidural disease (Bilsky grade). Also, there is room for refinement and validation of decision-making algorithms. Existing systems (NOMS, LMNOP, NESMS) need updating to reflect modern oncology. Research should integrate molecular and genomic tumor data to improve survival prediction. AI-enhanced, dynamic decision tools that incorporate imaging, lab data, and functional scores have to be devised and they need to be validated in international, multi-institutional databases for generalizability.

Biological therapies, including targeted therapies such as monoclonal antibodies and gene therapy, may offer synergistic effects when combined with MISS in controlling tumor growth and improving spinal stability [57]. Advances in cancer genomics and personalized medicine will allow for more tailored surgical approaches that consider the genetic profile of the tumor and the patient’s overall health condition [58]. Additionally, developing standardized protocols for enhanced recovery after MISS can further reduce recovery times and improve patient satisfaction.

## 7. Conclusions

Minimally invasive spinal surgery (MISS) has markedly transformed the management of metastatic spinal disease. By reducing operative trauma and facilitating faster postoperative recovery, MISS has contributed to lower morbidity and enhanced outcomes for patients facing spinal metastases. These advancements represent a significant evolution in oncologic care.

Algorithmic decision-making frameworks also play a crucial role in optimizing management. Such evidence-based tools help standardize clinical practice, reducing discrepancies across providers and institutions. Looking forward, refined decision-making systems should combine established principles with contemporary classification strategies to further improve care.

Ultimately, MISS exemplifies a patient-centered approach, delivering proven safety and efficacy. As this methodology is integrated alongside advanced radiotherapy and increasingly precise prognostic models, it forms the backbone of personalized, multidisciplinary cancer management. MISS is well-positioned to become the standard of care for appropriately selected patients.

## Figures and Tables

**Table 1:** Different techniques of minimally invasive spinal surgery.

	Advantages	Disadvantages
Small incision decompression	- This approach provides a minimally invasive option for 1–2 level procedures, reducing unnecessary surgical trauma. - It enables effective ventral decompression, which can significantly enhance outcomes when combined with SRS. - By establishing a protective margin around the dura mater, it facilitates safer and more effective adjuvant radiotherapy. - The combined strategy of surgery plus SRS—hybrid therapy—has demonstrated clear efficacy in clinical practice.	- Limited exposure, suitable only for 1–2 levels - Ventral decompression is technically challenging - Requires intraoperative validation with imaging/navigation
Percutaneous pedicle screw fixation	- Provides stabilization in an unstable spine - Can be used with small incision decompression - Cemented screws add reinforcement	- Screws are thicker and limited to lower thoracic/lumbar vertebrae - Not suitable as a standalone for all cases
Percutaneous vertebroplasty (PVP) or kyphoplasty (PKP)	- Alleviates pain while providing stabilization for microfractures - Demonstrates antitumor properties attributable to PMMA - Minimally invasive technique that supports the anterior spinal column - Permits concurrent biopsy during the procedure	- Less effective for posterior column involvement unless combined with pedicle screws
Endoscopy-assisted spine surgery	- Minimally invasive alternative in selected cases - Applicable in interlaminar and posterolateral approaches	- Narrow operative space - Difficult hemostasis - Steep learning curve
Radiofrequency ablation	- Minimally invasive and image-guided - Reduces pain and improves neurological function - Reduces fracture risk (when combined with vertebroplasty) - Enhances quality of life	- Not suitable in cases of spinal cord compression or instability - Contraindicated in infections and coagulopathy
Laser Interstitial thermotherapy (LITT)	- Minimally invasive, precise tumor ablation - Short recovery time - Preserves spinal stability and combines well with other therapies - Effective for thoracic epidural compression	- Limited to selected patients - Requires specific expertise and equipment
Radioactive seeds implantation	- Continuous radiation to the tumor without damaging the spinal cord - Effective pain relief and spinal stability - Can be combined with PVP/PKP - Improves quality of life and reduces paraplegia	- Requires precise planning and placement - Limited availability and expertise

**Table 2:** Comparison between open and MIS techniques.

Parameter	Open Posterior Fusion (OPF)	Minimally Invasive Spine Surgery (MISS)
Surgical duration [33]	Longer	Shorter (*p* = 0.035)
Blood loss [32,33]	Higher	Lower (418 mL less)
Transfusion rate [32]	41.3%	12.5% (*p* = 0.031)
Infection rate [32]	4%	1%
Hospital stay	Longer	Shorter
Mortality at 1 year [33]	21.4%	5.5%
Quality of life improvement (QLQ-BM22) [33]	Improved at 6 and 12 months	Improved at 6 and 12 months
Main limitation [32]	Applicable for patients needing decompression	Not suitable for decompression cases

**Table 3:** Therapeutic planning based on Bilsky grade.

Bilsky Grade	Description	Preferred Strategy (per NOMS/ESTRO)	Role of Radioresponsiveness
0–1a	Epidural tumor not deforming thecal sac	MISS stabilization ± SBRT (no decompression)	If radiosensitive: RT alone may suffice; MISS for pain/instability only.
1b–1c	Deformation of thecal sac but no cord compression	SBRT ± stabilization; consider MISS decompression if instability or refractory pain	Radiosensitive: SBRT alone; radioresistant: consider limited MISS separation surgery first.
2	Cord abutment without compression	Separation surgery + SBRT (hybrid therapy)	Radioresistant tumors (renal cell, melanoma, sarcoma) benefit most from hybrid therapy.
3	Cord compression with loss of CSF signal	Decompression mandatory—either open or MISS separation surgery → SBRT after healing	Radioresistant: critical to decompress before ablative SBRT; Radiosensitive: decompression only if incomplete response to RT.

**Table 4:** Definitive and relative indication for surgery.

Definitive Indication	Relative Indication
Clinical myelopathy due to metastatic epidural spinal cord compression	Asymptomatic metastasis in a structurally stable spine requiring biopsy or local control
Spinal instability—SINS ≥ 13, strongly supporting surgical stabilization	Intermediate SINS (7–12)—indeterminate stability; multidisciplinary discussion recommended to determine need for stabilization
Progressive neurological deficit despite adequate non-surgical therapy	Oligometastatic disease in a radioresistant tumor where durable local control is achievable
Intractable pain due to mechanical instability unresponsive to radiotherapy or medical management	Isolated radiculopathy from nerve root compression where decompression can improve quality of life
Need for tissue diagnosis when prior percutaneous biopsy is non-diagnostic	Patient preference or functional goals that justify limited intervention for pain or stability

**Table 5:** The New England Spinal Metastasis Score (NESMS).

Criteria	Modified Bauer Points	NESMS Points
Modified Bauer score components		
Primary tumor is not lung	1	
Primary tumor is breast or kidney	1	
Solitary skeletal metastasis	1	
No visceral metastasis	1	
Modified Bauer score		
	≤2	0
	≥3	2
Serum albumin (g/dL)		
	<3.5	0
	≥3.5	1
Ambulatory status		
	Non-ambulatory	0
	Intact or impaired	1

**Table 6:** Technique Matrix—Spine Metastasis (indications, contraindications, quick notes).

Technique	Typical Indications	Key Contraindications/Cautions	Quick Practical Note
MISS decompression/Minimally invasive separation surgery	- High Bilsky grade with cord compression requiring decompression but patient benefits from lower-morbidity approach. - Need to create ≥ 2–3 mm tumor-to-cord margin to enable SBRT (separation surgery). - Focal epidural disease at 1–3 levels where posterior access is sufficient and patient is frail or needs rapid recovery.	- Multilevel extensive disease requiring wide-field en-bloc resection. - Gross spinal instability that cannot be stabilized percutaneously (very complex constructs). - Poor visualization/heavy epidural vascularity when hemostasis cannot be achieved minimally invasively.	Use when goal = neural decompression + rapid recovery to permit adjuvant SBRT/systemic therapy; combine with percutaneous fixation if instability.
Cement-augmented pedicle screws (fenestrated screws + PMMA)	- Instrumentation in osteolytic metastatic bone, or poor bone quality (osteoporosis/prior radiotherapy). - Need for enhanced screw purchase in short-segment fixation for metastatic constructs.	- Active systemic or local infection. - Known allergy to PMMA components (rare). - Tumor with severe venous invasion or highly vascular bone where cement leak risk is unacceptable without mitigation. - Careful in pedicles with cortical breach—higher cement extravasation risk.	Cement augmentation improves pull-out and construct longevity but carry cement-leak and (rare) pulmonary embolus risk—use image guidance and staged/incremental cementing.
Radiofrequency ablation (RFA) ± vertebral augmentation (VAP: PVP/PKP)	- Localized painful vertebral body metastasis (pain refractory to meds). - Oligolytic lesion without major posterior column compression or gross instability. - As palliative local tumor control or to reduce tumor burden before/with cement.	- Spinal cord compression requiring decompression (risk of thermal cord injury). - Unstable vertebral body (unless combined with augmentation/fixation). - Active infection or uncorrected coagulopathy. - Lesion immediately adjacent to neural elements without safe ablation margin.	RFA gives rapid pain relief and is commonly paired with vertebroplasty/kyphoplasty to restore stability—must account for fracture risk after ablation.
Laser Interstitial Thermal Therapy (LITT/sLITT)	- Focal epidural metastatic compression where open decompression poses high surgical risk. - Small to moderate epidural tumor volumes (thoracic lesions reported often). - Patients who need minimal-access tumor cytoreduction to enable subsequent SBRT.	- Large-volume epidural disease requiring wide decompression. - Cases where thermal monitoring/cooling cannot reliably protect the cord (close, inseparable lesion). - Lack of center experience or unavailable MRI thermometry. - Active infection/coagulopathy.	LITT is promising as a minimally invasive ablative option for select epidural metastases; requires MRI thermometry and specialized team; consider as alternative to open decompression in selected patients.
Interstitial brachytherapy (I-125 seeds/CT-guided permanent seed implantation)	- Focal vertebral metastasis (pain control/local salvage) after prior EBRT or when EBRT is not feasible. - Small-to-moderate lesion volumes where conformal local dose is desired; often combined with vertebral augmentation. - Selected oligometastatic lesions for local control/palliation.	- Large, irregularly shaped lesions where dosimetric coverage is unreliable (edge miss). - Tumors intimately involving thecal sac/cord without safe margin or when seed placement would risk cord dose > tolerance. - Local infection/uncorrected coagulopathy. - Close proximity to hollow viscera or neurovascular structures if planning cannot respect constraints.	CT-guided permanent seed implantation (I-125) has shown pain relief and local control in selected series; careful pre-planning and post-implant dosimetry are essential. Often combined with vertebroplasty for mechanical support.

## Data Availability

Data is contained within the article.
